# Profile of suicide attempts and risk factors among psychiatric patients: A case-control study

**DOI:** 10.1371/journal.pone.0192998

**Published:** 2018-02-22

**Authors:** Meha Bhatt, Stefan Perera, Laura Zielinski, Rebecca B. Eisen, Sharon Yeung, Wala El-Sheikh, Jane DeJesus, Sumathy Rangarajan, Heather Sholer, Elizabeth Iordan, Pam Mackie, Shofiqul Islam, Mahshid Dehghan, Lehana Thabane, Zainab Samaan

**Affiliations:** 1 Department of Clinical Epidemiology and Biostatistics McMaster University, Hamilton, Ontario, Canada; 2 MiNDS Neuroscience Graduate Program, McMaster University, Hamilton, Ontario, Canada; 3 Bachelor of Health Sciences Program, McMaster University, Hamilton, Ontario, Canada; 4 Population Health Research Institute, Hamilton General Hospital, Hamilton, Ontario, Canada; 5 St. Joseph’s Healthcare Hamilton, Hamilton, Ontario, Canada; 6 Biostatistics Unit, Centre for Evaluation of Medicine, Hamilton, Ontario, Canada; 7 System-Linked Research Unit on Health and Social Service Utilization, McMaster University, Hamilton, Ontario, Canada; 8 Department of Psychiatry and Behavioural Neurosciences, McMaster University, Hamilton, Ontario, Canada; 9 Population Genomics Program, Chanchlani Research Centre, McMaster University, Hamilton, Ontario, Canada; 10 Peter Boris Centre for Addiction Research, St. Joseph’s Healthcare Hamilton, Hamilton, Ontario, Canada; Shinshu University School of Medicine, JAPAN

## Abstract

**Background:**

Suicidal behaviour remains challenging for clinicians to predict, with few established risk factors and warning signs among psychiatric patients.

**Aim:**

We aimed to describe characteristics and identify risk factors for suicide attempts among patients with psychiatric disorders.

**Methods:**

Multivariable logistic regression analysis, adjusted for clinically important confounders, was employed to determine risk factors for suicide attempts within a psychiatric patient population.

**Results:**

The case (n = 146) and control groups (n = 104) did not differ significantly with regards to sociodemographic characteristics. The majority of the participants who had attempted suicide did so with *high* intent to die, and expected to die without medical intervention. The primary method of attempt was pharmaceutical overdose among the case participants (73.3%). Results showed impulsivity (odds ratio [OR] = 1.15, 95% confidence interval [CI] = 1.03–1.30) and borderline personality symptoms (OR = 1.07, 95% CI = 1.01–1.13) were significantly associated with attempted suicide.

**Conclusions:**

Our findings indicate that known sociodemographic risk factors for suicide may not apply within psychiatric populations. Prevention strategies for suicidal behaviour in psychiatric patients may be effective, including limited access to means for suicide attempts (i.e. excess pharmaceutical drugs) and target screening for high-risk personality and impulsivity traits.

## Introduction

Suicidal behaviours are complex and can be challenging to foresee even among patients receiving medical and psychiatric care [1, 2]. Suicide is the second leading cause of death among 15–29 year olds worldwide [3], with an even greater prevalence of non-fatal suicidal behaviour [4]. Attempted suicide, defined as self-harm behaviour with intent to die [5], may occur up to 20 times more frequently than completed suicide [4, 6]. Attempted suicide is associated with adverse, long-term outcomes, including psychiatric and medical comorbidity, hospitalization, repeated suicide attempts, poverty, chronic stress, and stigma [7, 8]. Considering the personal and public health burden of suicide on global and local scales, it is necessary that preventative and rehabilitative strategies be developed to manage those presenting with suicidal behaviour.

While individuals with psychiatric illnesses represent the vast majority of the individuals who attempt suicide [4], only a small proportion of those with psychiatric illnesses attempt suicide. Known risk factors for suicidal behaviours are largely based on studies of general community populations and these factors include prior suicide attempts, underlying psychiatric and substance use disorders, single marital status, unemployment, and major life stressors [8–12]. However, reliable predictors of suicidal behaviour among populations with serious psychiatric disorders remain elusive. Wide-scale screening of psychiatric patients has been suggested as a method of early detection of suicidal behaviour [13], although feasibility greatly limits the ability to comprehensively screen all patients. Some studies have examined suicidal risk factors among patients with specific psychiatric disorders [14–16], yet suicidal risk factors among broad psychiatric populations who typically present to clinical settings, including patients with multiple psychiatric diagnoses, are not yet clearly established. Defining high-risk psychiatric patients will allow clinicians to effectively screen patients for suicidal behaviour.

Moreover, given the difficulties of identifying those at risk of suicide from populations of psychiatric patients, identification of warning signs and behaviours associated with suicide among psychiatric patients can aid in suicide prevention. Patterns and behaviours associated with suicide attempts are important to characterize within psychiatric populations, in order to distinguish individuals in this group who attempt suicide from those with no history of suicide attempts. Identifying trends in behaviours and methods of suicidal attempts within a sample of patients with psychiatric disorders will aid in the movement towards developing large-scale suicide prevention methods in clinical settings.

The present case-control study aimed to 1) describe the trends and circumstances associated with suicide attempts and 2) investigate risk factors of suicide attempts among adult inpatients with psychiatric disorders.

## Methods

The participants and data used in this investigation were collected for the Study of Determinants of Suicide Conventional and Emergent Risk (DISCOVER) [17], a case-control study designed to investigate risk factors of attempted suicide. Participants were recruited between March 2011 and November 2014 in Hamilton, Ontario, Canada. Data were collected at St. Joseph’s Healthcare and Hamilton Health Sciences hospitals. The study procedures were approved by the Hamilton Integrated Research Ethics Boards (HIREB) (REB number 10–661 for St. Joseph’s Healthcare Hamilton and 11–3479 for Hamilton Health Sciences Hospitals).

**Fig 1:**
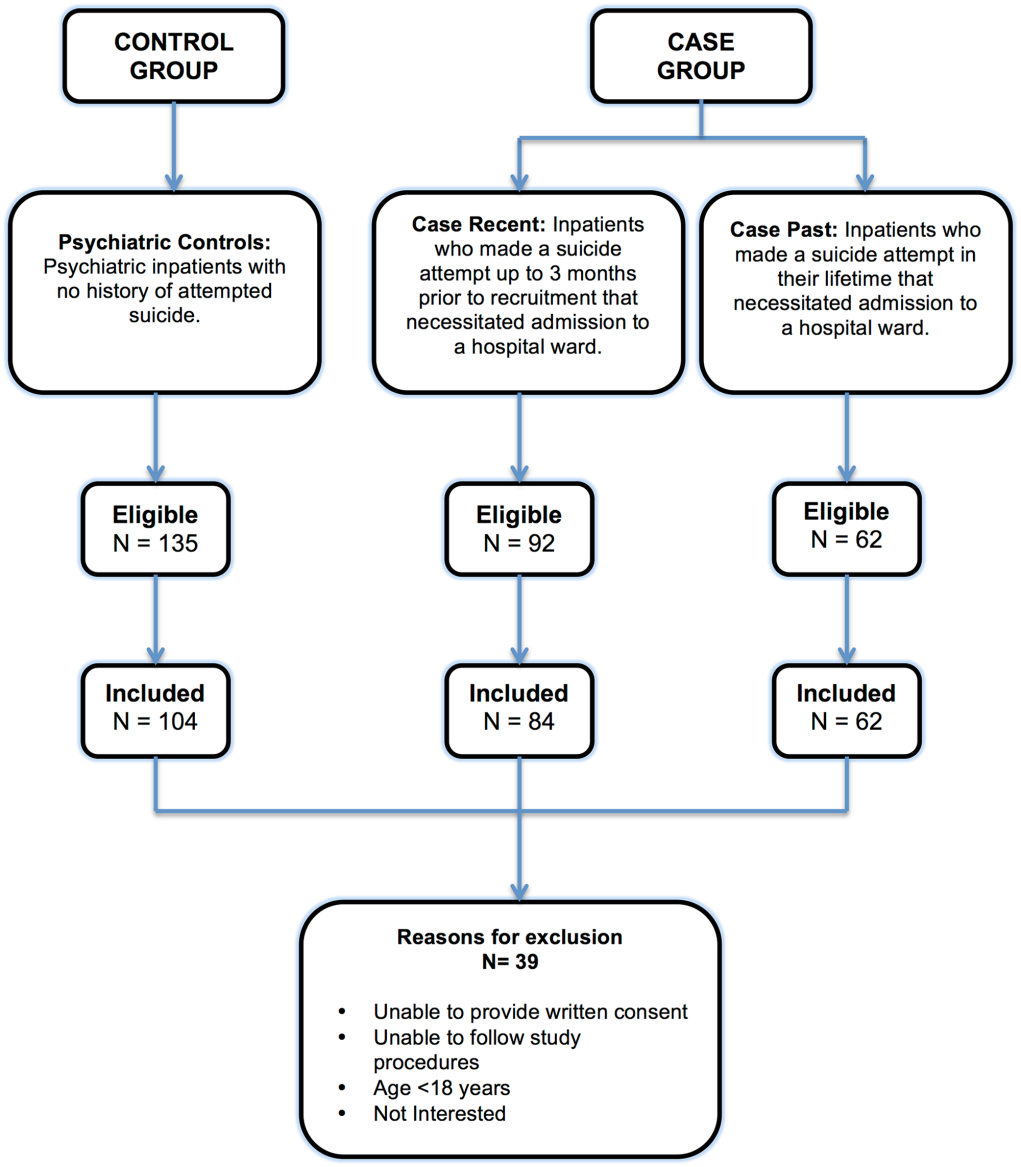
Eligibility and recruitment flowchart.

We included adults (≥ 18 years of age) who were able to provide written informed consent, communicate in English, and who were willing to follow study procedures. Fig 1 defines case and control groups and outlines the recruitment process. Cases included psychiatric inpatients who had made a suicide attempt, defined as self-directed injury with specific intent to die and that necessitated admission to a medical or psychiatric hospital ward. We had initially intended to recruit psychiatric inpatients who had made a suicide attempt within three months of recruitment (case-recent group). However, given the challenges of recruiting this particular patient population, we also included psychiatric inpatients who had a lifetime history of attempted suicide (case-past group). The control group consisted of adult psychiatric inpatients who had never attempted suicide and who were admitted to the same psychiatric hospital within the same time frame as the cases. The predefined study design included matching of case and control participants by sex and age (±5 years); however given the difficulties in recruiting case participants, matching was not implemented in order to reach our target sample size.

Clinical staff identified eligible hospitalized patients who had the mental capacity to provide written informed consent. These patients were consecutively approached by trained research staff who inquired about their interest in participating in the study and provided study information. Patients who agreed to participate were asked to read and sign consent forms describing study procedures. The local institutional ethics boards approved consent forms and procedures. Research assistants conducted a structured face-to-face interview consisting of validated questionnaires, described below. Participants were asked about sociodemographic characteristics including age, sex, education and socioeconomic status. We assessed intention to die as a result of the suicide attempt by asking the participants directly and measured level of intent in relation to participants’ most recent suicide attempt using the Pierce Suicide Intent Scale (P-SIS) [18]. The P-SIS consists of 12 questions assessing the circumstances during the suicide attempt, self-reported risk, and medical risk. The domain scores are tabulated to provide an overall assessment of level of intent to die as a result of a suicide attempt. This scale distinguishes self-harm behaviour from suicide attempts. The overall score on the P-SIS ranges from 0 to 25, with a maximum 3 or 4 points for each question, and higher scores corresponding to higher intent to die. Total scores of 0–3 represent *low* intent, 4–10 represent *moderate* intent, and 10 or more represent *high* intent to die. The scale has shown high test-retest reliability (r = 0.97).

Participants also completed the 30-item Barratt Impulsiveness Scale (BIS) [19] to assess trait impulsivity and the 23-item Borderline Symptom List (BSL) [20] to assess borderline personality symptoms. The BIS questionnaire was chosen to assess impulsivity as a risk factor independent of psychiatric diagnoses. The BIS measures impulsive behaviours on attentional, motor and nonplanning factors. Each question asks about an impulsive trait or behaviour on a 4-point Likert scale (Rarely/Never, Occasionally, Often, Almost Always/Always). The questionnaire has an overall maximum score of 120 and higher scores represent higher impulsivity, such that an increase of 4 points is indicative of an additional impulsive trait or behaviour. The questionnaire has shown good internal consistency (Cronbach’s α = 0.83) [19].

The BSL was used as a measure of borderline personality symptoms, with higher scores on representing increased severity of symptoms. Each question asks about a borderline personality symptom on a 5-point Likert scale (Not at all = 0, A little = 1, Rather = 2, Much = 3, Very strong = 4). The overall score is determined by dividing the sum of the individual item scores with the total number of items (overall score = sum/23 for this study). Although there is no clinical cut-off for diagnosis of borderline personality disorder on the BSL, researchers found a mean score of 2.05 (SD = 0.90) in a sample of borderline personality disorder patients. For the purposes of this study, we determined and reported the mean overall score for the case and control groups as described above, however we used the total sum of the individual questions as a continuous variable for the multivariable regression model (maximum score = 92). Therefore, a 4-point increase represented an additional borderline personality symptom. The questionnaire has shown good internal consistency (Cronbach’s α = 0.97) and test-retest reliability (r = 0.82, p<0.0001).

The Mini International Neuropsychiatric Interview (M.I.N.I.) [21] was administered to determine whether participants met criteria for DSM-IV Axis I psychiatric disorders. We used the M.I.N.I. to determine if participants had existing diagnoses of (1) mood disorders (major depressive disorder, bipolar disorder), (2) anxiety disorders (generalized anxiety disorder, panic disorder, social phobia, obsessive-compulsive disorder, post-traumatic stress disorder), (3) substance use disorders (alcohol dependence/abuse and substance dependence/abuse), and (4) psychotic disorders. The M.I.N.I. also determined if participants met the criteria for antisocial personality disorder. For this study, an experienced psychiatrist determined the participants’ primary Axis I psychiatric diagnosis using the hierarchical rules in the DSM-III [22] that were carried through to the DSM-IV classification system [23]. Therefore, the primary diagnosis was assigned using the following hierarchy: (1) substance use disorder, (2) psychotic disorder, (3) mood disorder, and (4) anxiety disorder.

STATA version 13 was used to perform all statistical analyses. For univariate analyses, we used independent sample t-tests to compare means of continuous variables and chi-square tests to compare proportions of categorical variables between cases and controls. Non-parametric equivalents (i.e. Mann-Whitney-U tests) were used for continuous variables that were not normally distributed. Fisher’s exact test was used to compare categorical variables that had an expected frequency of less than 5 in a particular cell. Simple Pearson’s correlations were used to assess the linear relationship between two normally distributed variables and Spearman correlations were used for non-normally distributed variables. Multivariable logistic regression analysis was utilized to assess clinical risk factors associated with suicidal attempts, by comparing cases to controls. The Hosmer-Lemeshow test was used to assess the goodness-of-fit of the regression model. Multi-collinearity between independent variables was assessed using the variance inflation factor (VIF), and variables with VIF>10 were considered for exclusion from the model. The level of significance was set at alpha = 0.05, and we included clinically important variables based on the literature regarding psychiatric populations in the logistic regression model [14–16, 24]. As a sensitivity analysis, we conducted multiple imputation using chained equations (MICE) to adjust for missing data in the multivariable regression model [25]. Age and sex were used to aid in the prediction of missing values in the imputed datasets. The reporting of this study is in accordance with the Strengthening of Reporting of Observational Studies in Epidemiology (STROBE) guidelines [26].

## Results

### Study sample characteristics

The final sample comprised 250 individuals, including 146 psychiatric inpatients who had attempted suicide (cases) and 104 psychiatric inpatients who had never attempted suicide (controls). Fig 1 displays the number of individuals approached for recruitment and included in the final sample as well as the reasons for exclusion. The sociodemographic characteristics of the case and control groups are summarized in Table 1. The mean age of the case group was 45.18 years (standard deviation [SD] = 14.69 years, range 18–73 years) and the control group was 45.01 years (SD = 14.23 years, range 18–82 years). The sample consisted of an approximately equal proportion of males and females (55.48% females in the case group, 50.00% females in the control group). In the univariate analyses, there were no significant differences in sociodemographic factors between cases and controls (p-values >0.05). The case group scored significantly higher on both BSL (p = 0.0003) and BIS (p = 0.0001) personality measures.

**Table 1:** Study sample characteristics.

	Cases (N = 146)	Psychiatric Controls (N = 104)	Univariate Test of Association
Age (years): mean (SD)	45.18 (14.69)	45.01 (14.23)	t = -0.09
			df = 248
			p = 0.928
Sex: n (% female)	146 (55.48)	104 (50.00)	χ^2^ = 0.73
			df = 1
			p = 0.392
**Education n (%)**			
Elementary	8 (5.56)	8 (8.0)	χ^2^ = 3.57
High-School	78 (54.17)	42 (42.00)	df = 2
Post-Secondary	58 (40.28)	50 (50.00)	p = 0.167
**Employment Status n (%)**			
Employed	34 (23.45)	33 (32.67)	χ^2^ = 2.56
			df = 1
			p = 0.110
**If Not Employed Then**:			
Unemployed	29 (26.61)	21 (32.31)	χ^2^ = 4.17
Retired	17 (15.60)	7 (10.77)	df = 3
On Disability	56 (51.38)	28 (43.08)	p = 0.244
On Social Security	7 (6.42)	9 (13.85)	
**Marital Status n (%)**			
Currently Married/Common Law/Live with Partner	39 (26.90)	31 (30.10)	χ^2^ = 5.38
Never Married	45 (31.03)	43 (41.75)	df = 2
Widowed/Separated/Divorced	61 (42.07)	29 (28.16)	p = 0.068
**Living Status n (%)**			
Lives Alone	64 (46.38)	47 (46.08)	χ^2^ = 0.0021
Doesn’t Live Alone	74 (53.62)	55 (53.92)	df = 1
			p = 0.963
**Smoking Status n (%)**			
Current Smoker	59 (43.38)	35 (35.35)	χ^2^ = 1.72
Former Smoker	34 (25.00)	26 (26.26)	df = 2
Never Smoked	43 (31.62)	38 (38.38)	p = 0.423
**Personality Measures**			
Borderline Symptom List Score: mean (SD)	1.82 (1.03)	1.32 (0.97)	t = -3.68
			df = 203
			p = 0.0003
Barratt Impulsivity Scale Score: mean (SD)	71.40 (12.03)	65.20 (10.23)	t = -4.14
			df = 213
			p = 0.0001

SD: standard deviation

Psychiatric diagnoses were assessed using the M.I.N.I for 211 participants and we reviewed medical records of 15 participants with missing M.I.N.I data to determine the psychiatric diagnoses. Information on the primary psychiatric diagnosis was missing for 24 participants. Table 2 presents the primary psychiatric diagnoses for the case and control groups. We utilized the primary psychiatric diagnosis, however 136 participants (60.17%) had a psychiatric comorbidity. Additionally, there were 20 participants in the case group and no participants in the psychiatric control group with a diagnosis of antisocial personality disorder although this was none of the participants’ primary diagnosis. Univariate analyses showed no differences in the prevalence of mood disorders, anxiety disorders, psychotic disorders or substance use disorders between the case and control groups.

**Table 2:** Primary diagnosis of psychiatric disorders (DSM-IV Axis I diagnosis).

	Cases n (%)	Controls n (%)	Univariate Test of Association
Mood Disorders	97 (74.6)	74 (77.1)	χ^2^ = 0.18
			df = 1
			p = 0.67
Substance Use Disorders (including Alcohol Abuse/Dependence)	19 (14.5)	11 (11.5)	χ^2^ = 0.48
			df = 1
			p = 0.49
Psychotic Disorders	11 (8.5)	6 (6.3)	χ^2^ = 0.39
			df = 1
			p = 0.53
Anxiety Disorders	3 (2.3)	5 (5.2)	p = 0.29*

*Fisher’s exact test was used to compare proportions due to low expected frequencies (<5).

**Notes**: Psychiatric diagnosis data was missing for 24 participants.

### Objective 1: Description of suicidal attempts and associated circumstances

Tables 3 and 4 summarize the methods and details of the suicide attempts (P-SIS). The majority of individuals attempted suicide by means of pharmaceutical overdose or pills (99/146). Other methods included cutting (25/146) hanging (7/146), suffocation (3/146), and firearms or explosives (2/146). There were 14 participants who reported using multiple methods during the suicide attempt.

**Table 3:** Method of suicide attempt.

	n
Pills	99
Cutting	25
Hanging	7
Suffocating	3
Firearm/Explosive	2
Other*	15

*Other methods include poisoning, drowning, setting self on fire, jumping from height, driving or jumping into traffic, alcohol consumption, starvation and intravenous drug use.

**Notes**: The total n is not in accordance with the sample size because 14 participants reported using multiple methods during the suicide attempt. 11 participants were missing data for method of suicide attempt.

**Table 4:** Suicide intent scale questionnaire in case group.

**Circumstances (N)**	**n (%)**
**Did you drink alcohol at the time of attempt? (120)**	
Yes	38 (31.67)
No	82 (68.33)
**Isolation: Was anyone around at the time of attempt? (131)**	
Somebody Present	25 (19.08)
Somebody Nearby or In Contact	34 (25.95)
No-One Nearby or In Contact	72 (54.96)
**Timing: (118)**	
Timed so that intervention is probable	38 (32.20)
Timed so that intervention is unlikely	38 (32.20)
Timed so that intervention is highly unlikely	42 (35.59)
**Precautions against discovery: (128)**	
No precautions	67 (52.34)
Passive precautions	26 (20.31)
Active precautions	35 (27.34)
**Acting to gain help during or after attempt: (131)**	
Notified helper regarding attempt	41 (31.30)
Contacted but did not specifically notify helper regarding the attempt	11 (8.40)
Did not contact or notify potential helper	79 (60.31)
**Final acts in anticipation of death: settling of affairs (130)**	
None	104 (80.00)
Partial preparation or ideation	13 (10.00)
Definite plans made (e.g. changes in will, taking out insurance)	13 (10.00)
**Suicidal note: Was a suicidal note left? (130)**	
No Note	95 (73.08)
Presence of Note	35 (26.92)
**Self-Report (N)**	
**Patient’s statement of lethality: Did you think that what you had done would kill you? (131)**	
Thought that what he/she had done would not kill him or her	24 (18.32)
Unsure whether what she/he had done would kill him/her	25 (19.08)
Believed that what she/he had done would kill him/her	82 (62.60)
**Stated intent: what was your intent at the time you made the attempt? (130)**	
Did not want to die	2 (1.54)
Unsure	21 (16.15)
Wanted to die	107 (82.31)
**Premeditation: did you consider attempting suicide prior to making the attempt? (130)**	
Impulsive, no premeditation	47 (36.15)
Considered act for approx. 1 hour	7 (5.38)
Considered act for approx. 1 day	8 (6.15)
Considered act for more than 1 day	68 (52.31)
**Reaction to act: how do you feel about the attempt now (130)**	
Patient glad she/he had recovered	62 (47.69)
Patient uncertain whether she/he is glad or sorry	44 (33.85)
Patient sorry he she had recovered	24 (18.46)
**Medical Risk (N)**	
**Risk predictable in terms of lethality of patient’s act and circumstances known: (126)**	
Survival certain	14 (11.11)
Death unlikely	33 (26.19)
Death likely or certain	79 (62.70)
**Would death have occurred without medical treatment: (130)**	
No	25 (19.23)
Yes	69 (53.07)
Uncertain	36 (27.69)

**Notes**: ‘N’ represents the total number of participants who responded to the question and ‘n’ represents the proportion of participants with the specified response.

The P-SIS assessed suicidal intent associated with the attempt based on circumstantial factors (e.g. alcohol consumption, suicide note), self-reported factors (e.g. patient’s beliefs regarding the lethality of the attempt), and medical risk (e.g. objective likelihood of death) (Table 4). According to overall P-SIS scores, 80.18% of the individuals who attempted suicide did so with *high* intent to die. Furthermore, 62.70% of the participants who attempted suicide believed that they would have died from the attempt and more than half of the individuals were found to have high medical risk for death based on objective questions. Of these individuals who attempted suicide, 47.69% reported feeling glad that they had recovered since the attempt.

### Objective 2: Risk factors for attempted suicide

Multivariable logistic regression analysis was used to determine factors associated with increased risk of attempted suicide. The model was adjusted for age, sex, and smoking status. Furthermore, variables were included in the model based on clinical importance and significant findings in the literature among similar psychiatric samples [24]. Diagnoses of mood disorders, psychotic disorders and substance use disorders were included, as well as personality measures, such as impulsivity and borderline personality symptom scores. The model indicated good fit, based on the Hosmer-Lemeshow test (p = 0.52).

The regression model demonstrated a statistically significant association between impulsivity and attempted suicide (odds ratio [OR] 1.15, 95% confidence interval [CI] 1.03–1.30, p = 0.02), as well as borderline symptoms and attempted suicide (OR 1.07, 95% CI 1.01–1.13, p = 0.02) (Table 5). The results suggest that an additional impulsive behaviour on the BIS is associated with 15% increase in odds of attempted suicide, and an additional borderline personality symptom on the BSL was associated with 7% increase in odds of attempted suicide.

**Table 5:** Risk factors for attempted suicide: Multivariable logistic regression results (n = 211).


Factors	Odds Ratio	95% Confidence Interval	P-value
Psychiatric Diagnosis			
Substance Use Disorder	Ref	Ref	Ref
Psychotic Disorder	1.28	0.33–4.96	0.72
Mood Disorder	0.91	0.37–2.25	0.85
Anxiety Disorder	0.37	0.06–2.16	0.27
Borderline Symptom Score	1.07	1.01–1.13	0.02*
Barratt Impulsiveness Score	1.15	1.03–1.30	0.02*
**Covariates**			
Age (years)	1.00	0.98–1.02	0.76
Sex: Female	1.47	0.81–2.66	0.21
Smoking Status: Current Smoker	1.29	0.69–2.41	0.42

*p-value<0.05

**Notes**: Age is interpreted as increase in one year. Borderline Symptom and Barratt Impulsiveness scores are interpreted as increases in four points on the questionnaires.

Given the comprehensive baseline interview, we expected missing data. Although we included 250 participants (146 cases, 104 psychiatric controls), 211 were included in the multivariable logistic regression due to missing data for psychiatric diagnoses, BIS, and BSL questionnaires. We conducted a multiple imputation as a sensitivity analysis to determine the impact of missing data on our results. The results were not changed in the imputed multivariable analysis, such that BIS scores remained significantly associated with an increased risk of attempted suicide (OR 1.14, 95% CI 1.03–1.27, p = 0.01) and BSL scores remained associated with an increased risk of attempted suicide (OR 1.06, 95% CI 1.01–1.12, p = 0.03). Furthermore, age, sex, smoking status and all primary psychiatric diagnoses were not associated with attempted suicide (p-values >0.05).

## Discussion

We sought to summarize characteristics and behaviours of adults associated with suicide attempts, which may help classify factors influencing suicidal behaviour within a clinical setting. We further aimed to make comparisons between individuals who had attempted suicide and a control group of psychiatric inpatients with no history of suicide attempts to identify risk factors among a vulnerable population. The findings indicated that adult psychiatric patients who had attempted suicide did not significantly differ from the psychiatric control group on sociodemographic characteristics or the prevalence of psychiatric diagnoses. The case group showed higher prevalence of maladaptive personality measures such as impulsivity, borderline symptoms and diagnosis of antisocial personality disorder. Factors such as single marital status, unemployment and low education level did not significantly differ between the case and control groups in this study, but have been reported as significant risk factors in studies comparing cases to community controls [4, 8, 9]. These findings suggest that known risk factors of suicidal behaviour may not be applicable within psychiatric inpatient populations, further confirming the necessity of identifying risk factors within psychiatric patients.

### Behaviours associated with suicide attempts and implications on prevention

Identification of trends and behaviours such as suicidal attempt methods, potential precautions against discovery, and feelings related to recovery may be helpful in elucidating warning signs and prevention strategies of attempted suicide within this population. The most common method of suicide attempt was ingesting pills or pharmaceutical overdose (72.26%) in this study. This may have been due to greater accessibility to medications in this sample, considering that participants had psychiatric comorbidities, as well as a modest prevalence of self-reported chronic pain (29.4%) and alcohol (21.2%) or substance use disorders (13.6%). The literature shows that access to methods of suicide may increase the risk of completed suicides [27–30] and limiting access to lethal methods can be an effective prevention strategy [31]. A large ecologic study on survey-based data from the United States found higher rates of firearm suicides in states with high gun ownership, while non-firearm related suicides were equal across the states [30]. Similar results have been found in studies of adolescent suicides examining access to firearms in the household [32, 33]. An analogous trend may follow among psychiatric populations with access to high doses of pharmaceutical drugs. Furthermore, research in the United Kingdom exploring limited access to over-the-counter pain relievers found that changing legislation to reduce pack sizes and enforcing a limit in purchasable tablets led to significant decreases in documented overdoses of these drugs over the following years [28]. These strategies may be important to consider among Canadian psychiatric populations as a recent study in Toronto found that among cases of completed suicides by overdose, prescription medications (including opioid analgesics and psychotropic drugs) were involved in the majority of suicides [34].

Evaluation of behaviours related to the suicide attempt revealed that almost half (45.03%) of the individuals attempting suicide had somebody “present” and “nearby or in contact” at the time of the suicide attempt. Furthermore, more than half (52.34%) the individuals who attempted suicide took no precautions against discovery. This demonstrates the importance of educating the families and friends of at-risk psychiatric patients regarding warning signs and communication strategies. However, identification of warning signs remains challenging given that a large proportion (80.00%) of individuals attempting suicide took no final actions in anticipation of death (i.e. settling of affairs), which has been previously reported as a warning sign for suicidal behaviour [13]. Approximately one-quarter of the participants who had attempted suicide left a note. Studies of individuals who completed suicide report a widely varying proportion of suicide notes from 18% to 37% and show that youth are more likely to leave notes [35].

The current study sample also showed that most cases of suicide attempts had serious intent to die and the majority of patients believed that the attempt would lead to death. However, the proportion of individuals attempting suicide with high intent to die in this sample appears greater than other samples, in which half of the participants report that they had low intent to die or attempted as a “cry for help” [10]. This finding may be related to the higher severity of psychiatric illness in this sample, since all participants were psychiatric inpatients. While the majority of participants reported high intent to die at the time of attempt, 47.69% of patients who had attempted suicide stated they were “glad that they recovered”. Given that a history of attempted suicide is among the strongest predictors of completed suicide [3], it is important to conduct future research among individuals reporting ambivalence related to physical and psychological recovery, as this may play a role in the prevention of repeat suicide attempts.

### Personality variables associated with increased suicide attempt risk

Trait impulsivity and borderline personality symptoms were statistically significant risk factors for attempted suicide, when compared to psychiatric controls. ASPD was only present in the case group, therefore it was completely associated with attempted suicide and was not included in the adjusted regression model. Other studies have found a significant association between antisocial personality symptoms and increased risk of attempted suicide [8, 36]. Within this sample of psychiatric patients including participants with multiple psychiatric diagnoses, mood disorders, substance use disorders, and psychotic disorders were not found to be significantly associated with attempted suicide. This may be due to the similar prevalence and high severity of psychiatric disorders in the case and control groups, since included participants were all hospitalized patients. Findings for impulsivity and borderline personality symptoms are consistent with previous studies among psychiatric populations [37, 38], though some of these studies were conducted among patients with specific psychiatric disorders, such as major depression [39] or bipolar disorder [40].

Interestingly, trait impulsivity was associated with increased odds of attempted suicide yet no significant correlation was found between trait impulsivity and the self-reported impulsiveness of the suicide attempt. These findings suggest that trait impulsivity may not necessarily drive individuals to make impulsive suicide attempts. Baca-Garcia et al. found similar results when exploring the relationship between trait impulsivity and suicide attempt impulsivity [41]. This may have clinical implications in increasing the feasibility of suicide attempt prediction among psychiatric patients, given the challenges associated with prevention of impulsive attempts. Additionally, these results suggest that development of clinical screening tools for trait impulsivity and borderline personality symptoms among psychiatric patients may be important in suicide attempt prevention.

### Limitations

This study was limited by potential biases due to self-report measures as well as the case-control study design. Within the case group, the case-past group may have been differentially affected by recall bias related to the details of the suicide attempt. Furthermore, the cross-sectional design of the study did not allow us to determine the direction of the association risk factors and suicide attempts. For example, suicide attempts, particularly in the case-past group, may have occurred before the manifestation of borderline personality symptoms. However, it was expected that the measurement of trait impulsivity would remain fairly constant over time. Future prospective research among psychiatric inpatients is needed to identify factors than can be predictive of future suicide attempts. It is also important for future studies to collect information about specific pharmaceutical drugs or “pills” used to attempt suicide among psychiatric patients, as this may aid in developing prevention strategies.

It is also important to note that the clinical characteristics and risk factors in this study apply to psychiatric patients who attempted suicide. This may not be generalizable to psychiatric patients at risk for completed suicide, as differences in risk factors between those who attempt and complete suicide have been reported in the literature [42]. Studies comparing risk factors for completed suicide, specifically within psychiatric patient populations, are warranted and can be used in conjunction with suicide attempt risk factors to develop an overall framework for suicide prevention among patients with psychiatric disorders [43].

## Conclusions

Findings from this study indicate that those who attempt suicide may not differ significantly from psychiatric inpatients on sociodemographic factors such as unemployment, single marital status and living alone. Impulsivity and borderline personality symptoms were found to be risk factors for suicide in this sample of psychiatric patients. A descriptive assessment of suicide attempts indicated that limiting access to methods of suicide and educating social supporters of hospitalized psychiatric patients regarding behavioural trends may be effective suicide prevention strategies. Future qualitative research can identify themes associated with thought processes leading to suicide attempts and feelings related to recovery among high-risk psychiatric patients. Additionally, cohort studies among patients with serious psychiatric disorders (i.e. psychiatric inpatients) are needed to establish the temporal association between personality variables and attempted suicide and to further identify unique risk factors for suicide attempts in psychiatric populations.

## Supporting information

S1 File Dataset. (XLSX)
